# Interventions to reduce social isolation and food insecurity in older adults: a systematic review

**DOI:** 10.3389/fnut.2025.1607057

**Published:** 2025-06-06

**Authors:** Keya Sen, Clemens Scott Kruse, Michael Mileski, Zo Ramamonjiarivelo

**Affiliations:** School of Health Administration, Texas State University, San Marcos, TX, United States

**Keywords:** food insecurity, social isolation, malnutrition, technology, intervention

## Abstract

**Introduction:**

Food insecurity (FI) and social isolation (SI) are interconnected social determinants of health that disproportionately affect older adults. While FI and SI have been studied independently, their combined effects on malnutrition and quality of life (QoL) have not been adequately synthesized. This systematic review aimed to evaluate the efficacy of combined FI and SI interventions over the past decade, identify reported facilitators and barriers, and explore technology-based approaches to inform future research.

**Methods:**

This study follows the Preferred Reporting Items for Systematic Reviews and Meta-Analyses (PRISMA) 2020 guidelines and the Kruse Protocol. Peer-reviewed empirical studies were identified through systematic searches of PubMed, Web of Science, CINAHL, and ScienceDirect. Inclusion criteria targeted non-randomized controlled trials and community-based interventions focused on adults ≥ 65 years in the past 10 years. Six studies were examined using a narrative approach, supported by data matrices.

**Results:**

The studies suggested a relationship between FI and SI, among individuals with physical impairment, low income, and reduced access to community resources. Women were most affected. Although the studies were observational and varied in quality, findings indicated that FI and SI are associated with adverse health outcomes, such as depression, diabetes, cognitive decline, and reduced QoL. Promising interventions included commensality programs, food service apps, and technology supported community engagement, though barriers such as limited digital literacy, high costs, and infrastructure gaps persist.

**Conclusion:**

The review underscores the need for integrated interventions as the global older adult population grows. Due to limited methodological rigor, definitive conclusions cannot be drawn. Future research should use robust designs-randomized controlled trials, mixed methods, and longitudinal studies, and address structural barriers, including digital exclusion, to improve health outcomes.

**Systematic review registration:**

https://www.crd.york.ac.uk/PROSPERO/view/CRD420250418740.

## 1 Introduction

While the consequences of both Food Insecurity (FI) and Social Isolation (SI) are known to have negative impacts on the physiological and psychological health of older adults age ≥ 65 years, the intersection of FI and SI is largely an unresearched area. FI is a problematic term to define. It can be described as a condition characterized by limited access to nutritious food, uncertain availability of food, and lack of socially acceptable means to obtain food (1). It also contributes to malnutrition, a condition that arises when a person’s diet lacks sufficient nutrients or contains too much of certain nutrients, leading to health problems (2, 3). SI is an objectively observable condition that exacerbates the psychosocial difficulties individuals experience due to a lack of social proximity and engagement, even if they do not subjectively feel lonely (4). The rising rate of FI combined with a higher risk of SI associated with increasing age disproportionately affects the quality of life of older adults (1) and can be addressed through nutrition support programs. For older adults, age-related physical comorbidities, the death or loss of friends and family, and impairments in cognition and sensory systems limit the frequency of social interaction. This in turn, reduces access to resources (5) information about quality food (6) and awareness of food services available within the community (7).

SI and FI are interrelated social determinants of health that frequently co-occur and influence one another, particularly in older adult populations. This relationship can be understood through an ecological or social determinants framework (8), which emphasizes that health outcomes are shaped by a dynamic interaction across multiple levels: individual, interpersonal, community, and societal. At the individual level, factors such as age, gender, race/ethnicity, income, education, health status (including depression, BMI, and limitations in daily activities), and length of time in the U.S. can directly influence an older adult’s vulnerability to both SI and FI. Moving to the interpersonal level, the presence or absence of emotional and financial support networks, such as family, friends, or caregivers can either buffer against or exacerbate the risks of isolation and limited food access. At the institutional level, access-related challenges—such as lack of private insurance, limited routine healthcare, and long travel distances to grocery stores—can further restrict food availability and deepen social disconnection. Community-level influences also play a significant role. Factors like rural versus urban residence, regional geographic differences, and the availability of community-based resources (e.g., meal delivery programs or senior centers) can support or hinder both social engagement and nutritional well-being. Finally, at the policy and societal level, broader structural elements, especially access to food assistance programs like the Supplemental Nutrition Assistance Program (SNAP), are crucial in shaping outcomes and reducing disparities related to SI and FI.

When considering FI, approximately 6.9 to 8.3% of the United States’ older adult population are at risk due to economic decline (9, 10). In fact, FI and malnutrition have associations with many diseases which affect older adults. More precisely, older adults experiencing FI not only have low nutrient intake, but also suffer from poor health, depression, diabetes, obesity, and functional limitations (11–13). Studies also posit that cognitive function can be negatively affected by FI and malnutrition (14–18). Furthermore, there is evidence that cardiometabolic risk may be present in those exposed to FI (19).

A population-level intervention addressing FI and SI for older adults would involve a policy or program designed to improve access to food, through community-based initiatives that simultaneously foster social connection and reduce feelings of isolation. These include community gardens, community kitchens, congregate meal programs, or social support networks linked to food distribution programs (20). Key elements of such intervention involve connecting people to resources such as screening for FI, providing referrals to food assistance programs, like Supplemental Nutrition Assistance Program (SNAP) proving free or low-cost meals, and building skills and confidence through nutrition education. These programs aim to enhance community food security and social support by implementing “check-in” systems within food distribution programs to ensure regular contact with vulnerable individuals. For older adults, partnering with local community centers or senior centers for food accessibility alongside social activities are effective as individuals connect while assisting with food distribution and consumption based upon culturally appropriate and local needs.

Some key interventions that attempt to address FI and SI in older adults include eating together or commensality, a health-promoting activity that contributes to improved health outcomes (21) with enhanced social support supplemented by dietary intake in multiple ways (22). Community-organized activities, such as Food Classes for Older Adults (FCOA), can provide sustainable commensality for homebound older adults who are often unable to cook for themselves (22.7%), those who are unable to shop for themselves (31.4%), and those who (14.6%) report money as a concern (23). When such programs are supported by apps or technology, they are more effective, as technology-based interventions such as mHealth provide the opportunity to stay socially connected (24) thus mitigating SI, and reducing the adverse impacts of FI (25).

Internet Food Delivery Applications (IFDA) are one way to mitigate the psychological issues of loneliness or depression and redress SI in older adults. Connecting to older adults, the service providers of IFDA optimize and enhance the nutrition status and deepen their understanding of how the issues of SI can factor into the pathophysiology of older adults as well. While services of this kind are common in some places of the United States, some regions are still in dire need. For example, in central Texas, there is a significant population of underserved, poor, insecure, malnourished, and socially isolated older adults (26, 27). Given that these measures are important social determinants (28) of health, programs that address these issues are necessary. Texas is ranked 44th in the United States for those aged 65 and older in poverty with an overall state poverty rate of 10% (26). With 33% Hispanic population, nearly 19% of the population aged 60 and older report food insecurity (29). The average malnutrition crude death rate in Texas is 65.6 per 100,000 before the COVID-19 pandemic based on 2014–2018 American Community Survey data (30), which worsened during the pandemic as malnutrition is a risk factor for COVID-19 mortality (29, 31).

The purpose of this systematic review was to identify and analyze the efficacy of combined SI and FI interventions for older adults over the past 10 years and analyze the facilitators and barriers in the published literature. We also attempted to review studies that implemented technology-based interventions and collected insights about FI and SI that might lead future researchers to additional interventions.

## 2 Materials and methods

### 2.1 Protocol and registration

This review was conducted in accordance with the Kruse Protocol (32) and reported in accordance with the Preferred Reporting Items for Systematic Reviews and Meta-Analysis (PRISMA, 2020) (33). The review is registered with PROSPERO (ID: CRD420250418740).

### 2.2 Inclusion and exclusion criteria

The criteria used to choose the articles for this systematic review consist of peer-reviewed empirical studies (non-clinical trials) and community programs/interventions that address FI, SI, or malnutrition with respect to the older adults ≥ 65 years old, over the last 10 years, regardless of geographic boundaries.

This study excluded randomized controlled trials (RTC), studies on interventions that targeted individuals < 65 years old, and systematic reviews published before 2012 (to prevent confounding results). This was done because systematic literature reviews already reported results from studies that may have been included in our analysis.

### 2.3 Information sources

Authors queried four databases: PubMed (MEDLINE), Cumulative Index to Nursing and Allied Health Literature (CINAHL), Web of Science, and ScienceDirect. These four databases were chosen because of their focus on health and nutrition research. Also, they are readily available to most researchers, and they are exhaustive in their content. MEDLINE was excluded from all but PubMed to help eliminate duplicates.

### 2.4 Search strategy

Starting with the key terms from the articles used in the introduction, a Boolean search string was created. We vetted these terms in the Medical Subject Headings (MeSH) of the U.S. Library of Medicine. Our final search string was: (“food insecurity” OR “nutrient insufficient” OR “food supply”) AND (“older adult” OR “elderly” OR “senior adult” OR “mature adult”) AND “social isolation.” We used the same search strategy in all databases and used similar filter strategies because not all databases utilize identical tools.

We included the term “intervention” in the Boolean search string: (“food insecurity” OR “nutrient insufficient” OR “food supply”) AND (“older adult” OR “elderly” OR “senior adult” OR “mature adult”) AND “intervention” AND “social isolation,” in our search for published articles within the last 10 years, and we could not find any articles (zero articles). It was only after we removed the term “intervention” that we were able get the 392 articles which the study was based upon. This shows the need for research regarding interventions in this area.

### 2.5 Selection process

**Figure 1:**
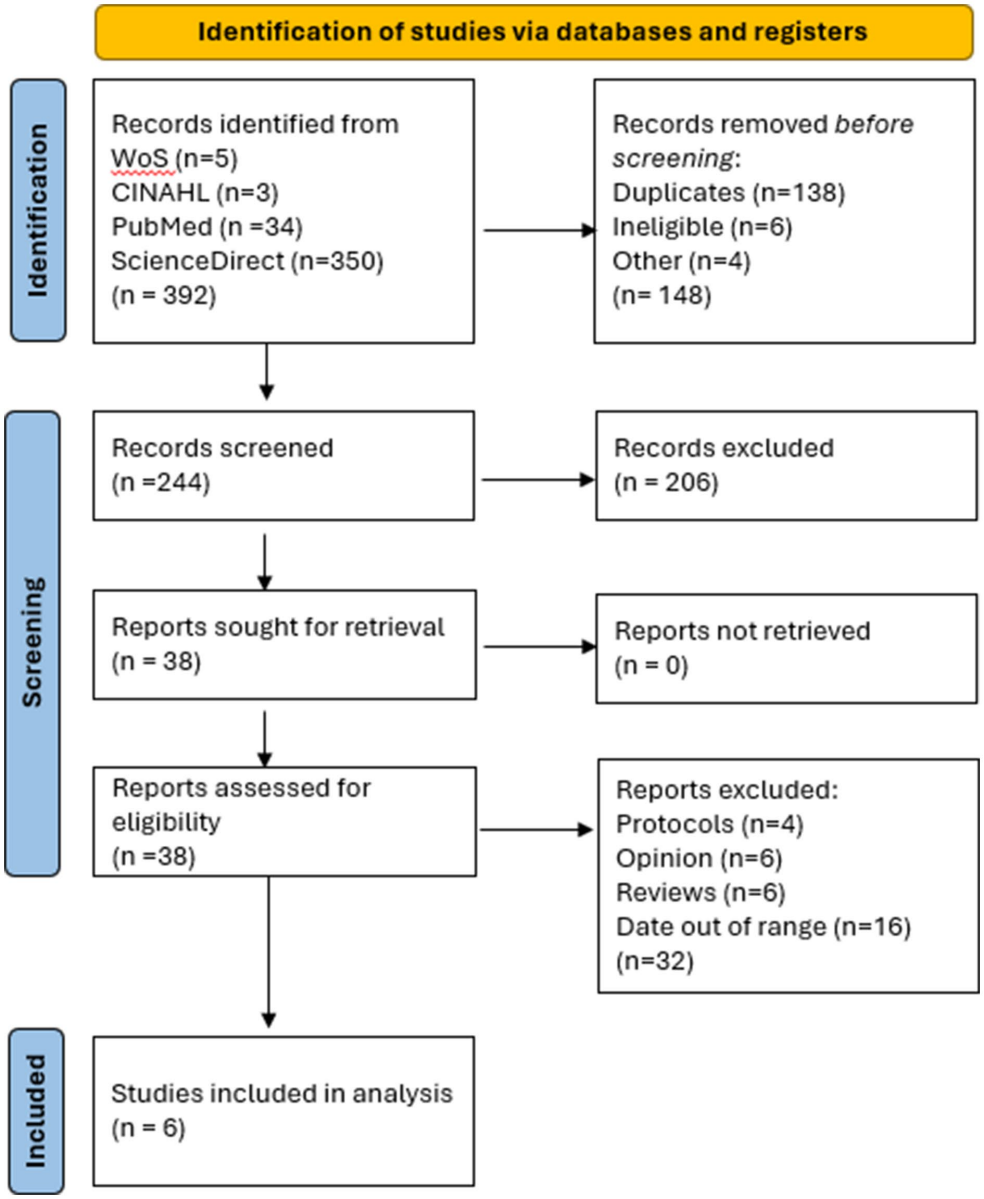
Flow diagram of study selection procedure according to PRISMA 2020.

This study followed the Kruse Protocol for systematic literature that consists of a series of three consensus meetings among the authors. The first consensus meeting consists of finding the articles that met the inclusion criteria and assessed their relevance to the topic and calculating the Kappa statistic of agreement. The second consensus meeting involved summarizing articles using a literature matrix manager in Excel, which required searching for identical key terms across all databases. After conducting the searches and filtering the results (34), we screened the abstracts for relevance and calculated a kappa statistic to assess agreement. To ensure that each abstract was screened by at least two observers, the project leader assigned an agreed-upon workload. Thus, we created the flow diagram of study selection procedure (Figure 1) (33).

### 2.6 Data collection process

Data was extracted using an Excel spreadsheet standardized by the Kruse Protocol. We analyzed each article and grouped the observations. Weekly consensus meetings were used over a period of 7 months to ensure a continuity of process which included both the data extraction and full analysis steps.

### 2.7 Data items

Standard fields were collected, as identified by the Kruse Protocol: From the Google Scholar step, we collected date of publication, authors, study title, journal, impact factor from Journal Citation Reports, study design, key terms, experimental intervention, results, and comments from each reviewer; from the article filtering step, we collected the number of results before and after each filter applied in all four databases, the filters used, and the articles excluded by each filter. In the abstract screening step we collected database source (MEDLINE, CINAHL, Web of Science, and ScienceDirect), date of publication, authors, study title, journal, screening decision for each reviewer, notes about rejections, consensus meeting one, determination of screening decision, and a set of rejection criteria; Finally, in the analysis step we collected database source, date of publication, authors, study title, participants, experimental intervention, results compared with a control group, medical outcomes, study design, sample size, bias effect size, country of origin, statistics used, the strength and quality of evidence, effectiveness of intervention, general observations about FI and SI, facilitators to adoption, and barriers to adoption (32). Most of these fields are standardized on the Excel spreadsheet, but the last four items were specific to the research objective.

### 2.8 Effect measures

The preferred measure of effect was the Cohen *d*, however, the only measure of effect reported in the articles kept was the Odds Ratio. Because we accepted mixed methods and qualitative studies, we were unable to standardize summary measures, as would be performed in a meta-analysis. Measures of effect are summarized in tables for those studies in which it was reported. The *kappa* statistic was calculated and reported (35, 36).

### 2.9 Synthesis methods

The Kruse Protocol for conducting a systematic literature review typically includes a thematic analysis, commensurate with techniques used in the American Psychological Association (37). Observations were grouped into themes, and themes were organized into affinity matrices for further analysis.

### 2.10 Additional analyses and certainty assessment

During the data-extraction step, effect sizes were calculated and tabulated. We combined the observations and effect sizes for the certainty assessment. Themes and observations were sorted based on frequency in affinity matrices. This does not imply importance: this action only provides the probability of encountering a theme or observations in the group of articles selected for analysis.

## 3 Results

The flow diagram of study selection procedure (Figure 1) shows that a total of 392 results were provided by the four research databases from our search string, of which 138 were duplicates. A total of 244 articles were screened. Screening removed 206 results, leaving 38 full-text studies to be analyzed. A Cohen kappa was calculated from this process (*k* = 0.95, high agreement) (35, 36). During the analysis phase, an additional 32 studies were removed (not caught in the abstract screening process). This left 6 articles for full analysis.

### 3.1 Study characteristics

Following the PRISMA 2020 checklist, we extracted data fields for each study: participants, intervention, comparison (control or other group), outcomes, and study design or statistics (Table 1). We found six studies published in the following years: 2013 (38), 2016 (39), 2017 (40), 2018 (41), 2021 (42), and 2022 (43). All studies involved older adults (over 60 years), and all reported elements of FI, SI, or malnutrition. Table 1 summarizes the study characteristics in a manner established in the literature (44).

**Table 1:** Participants, intervention, results, outcomes, and study design.

Authors	Participants (demography of group analyzed)	Intervention type	Results (compared to control group), what did they find?	FI and SI reported	Study design
Simsek et al. (38)	Older adults aged 65–97 (mean age = 74.1); Participants 62.9% female (n = 409).	Outputs were based on demography. No specific intervention reported.	No control group. Sample percentage with: FI prevalence = 21.7%, malnutrition prevalence = 2.7% and malnutrition risk = 28.0%.	FI was reported. SI was totally ignored.	Cross-Sectional
Vilar-Compte et al. (39)	Older adults ≥65 in Mexico City as part of a community group for senior citizens	No specific program or experimental design was found.	No control group. Primary education and receiving cash-transfers (OR = 0.478) were significantly associated with a lower probability of being moderately-severely food insecure (OR = 0.597). The probability of moderate-to-severe FI was significantly higher among older adults at risk of depression (OR = 2.843), those with at least one activity of daily living impaired (OR = 2.177) and those with at least one instrumental activity of daily living impaired (OR = 1.785).	FI was reported. SI in connection with FI was not reported	Mixed Methods
Ishikawa et al. (40)	Older adults between 65–90 years, 66% female (63.5% response rate).	Outputs were based on observation. No intervention type reported.	No control group. 47.1% of men and 23.9% of women ate together less than once a month. Those who ate together less than once a month had a significantly lower rate of subjective health, food diversity, and food intake frequency than those who ate together more often. A stepwise logistic analysis showed that the factors most strongly related to eating together less than once a month were not having any food shopping assistance	Significant connection between FI and SI was found. Men who ate together were more satisfied with diet; women who ate together felt healthier, were more satisfied with diet, and had a wider range of foods.	Mixed Methods
Ganhao-Arranhado et al. (41)	Older adults ≥65, average age 78.4, 62.3% female	Reports were based on a program, not concluded as intervention	No control group. Contributors to risk of malnutrition or nutrition were women, FI, reported depression, loneliness, acute myocardial infarction, cerebrovascular accident, diabetes, age 74–85, health status and attending senior centers for less than 5 years. FI resulted from low monetary status, socialization was a reason for attending senior centers, social risk, and diabetes. There was no correlation between FI and obesity, but positive correlation existed between FI and weight.	The Relation between FI and SI was established. 70% were FI, 40.7% at risk of malnutrition, 4.7% malnourished, 34.7% high social risk	Observational
Mehran et al. (42)	Older adults ≥65, average age 79.3, 58% female, 55% Caucasian (White)	Linking patients facing FI to a food assistance program called Fresh Food Rx program during COVID 19 pandemic. Telehealth assessed safety, FI, SI and loneliness	No control group. Participants who screened for FI (23%) related to the intervention—no further measurements were collected. Results from survey indicated: 23% (11/48) of survey respondents experience food insecurity; 8% (4/48) have caregiver issues; 8% (4/48) need medical supplies, 11/48 (23%) are in need medications	The survey did not cover the relationship between FI and SI	Non-experimental (no randomization, no control)
Hashemi et al. (43)	Older adults >65	DASH at a community senior center	No control group. DASH-aligned meals showed rapid improvement in hypertension among participants.	FI and SI are addressed through a community senior center (4 meals per week). Blood Pressure decreased by 1 month.	Pre-post

SI, Social Isolation; FI, Food Insecurity; OR, Odds Ratio; Rx, Medical Prescription; DASH, Dietary Approaches to Stop Hypertension.

### 3.2 Results of individual studies

Reviewers recorded the observations in Table 2 from each article in accordance with the objective statement. Several studies in this review suggest an association between FI and SI, particularly noting that lack of access to community programs contributes to both limited food availability and reduced social support for older adults living alone with functional limitations and limited resources. People who ate together, which means people with social or community support, had less SI and more food security. A thematic analysis was conducted to make further sense of the information collected from the articles (37). Articles listed in this study used various themes to summarize the results and observations, but these themes did not always match the authors’ observations exactly. These themes can be seen in the affinity matrix in Table 3. The thematic analysis helps make sense of the data extracted. When an observation re-occurred, it became a theme. However, due to the small number of articles surrounding the topic, observations (themes without recurrence) were also included in the affinity matrix.

**Table 2:** Summary of analysis.

Authors	Effectiveness of intervention	General observations on FI and SI, and malnutrition	Facilitators to adoption	Barriers to adoption
Simsek et al. (38)	N/A	Malnutrition was determined by using MNA tool. Food insecurity was defined by the NSENY. Data was collected at home via face-to-face interview and by measuring anthropometric indices. SI was not reported. Factors that are positively associated with malnutrition and risk of malnutrition were age, number of chronic diseases, not being married, being poor-to-very poor, having orthopedic disability, having food insecurity, and poor very poor self-perceived health. Causality inference does not apply (cross-sectional survey data).	N/A	N/A
Vilar-Compte et al. (39)	N/A	Higher education and cash transfers have a positive influence on reducing food insecurity. Depression and functional limitations increase the likelihood of FI. Mostly older female urban adults were studied. Increased education and increased cash led to increased access to community programs and decreased concerns with FI. Lack of money and lack of access to community programs was associated with FI. It correlated with concerns with functional limitations, as well as a lack of pension or social security. Socioeconomic resources, basic health concerns, built environment, and SI are concern. Reports on marginal effects of social programs, functional impairments, health status, mental health, SI, FI and food availability in Mexico City. Marginal effects analysis indicated that some basic education associated with low probability of FI compared to having no education.	Increased education, increased cash/assistance, increased access to community programs	Lack of cash, lack of access to community programs
Ishikawa et al. (40)	N/A	Eating together was positively correlated with subjective health in Japanese people living alone. Those who live alone had concerns with food behaviors and FI. Inadequate nutrient intake was correlated with eating in isolation, especially in men. Food accessibility is active behavior and having others around increased nutrition. Men and women who ate together were more satisfied with diet; felt healthier, had more social connectedness. Eating together increased satisfaction, eating frequency, food diversity, and food intake. Eating alone or less eating together with people equate to lower subjective health, food diversity, and increased food intake frequency. While describing behavior (togetherness),only SI was reported, FI was not reported. Men who ate together less than once in a month were 3.06 times more likely to not have someone to help them with food shopping, 1.74 more likely to not receive any food from neighbors or relatives, and 2.16 times as likely to be of low income.	Eating together increases satisfaction, eating frequency, food diversity, and intake.	Less eating together equates to lower subjective health, food diversity, and food intake frequency.
Ganhao-Arranhado et al. (41)	N/A	Malnutrition was measured by MNA, Food insecurity was measured by FIES, Social risk measured through Gijon’s social-familiar evaluation scale. Nutritional status and FI were associated with health status and social circumstances, such as diabetes, loneliness, and lack of economic resources. Those who reported depression were 37.41 times as likely to experience FI, in comparison to those who did not report depression. Females were 7.87 times as likely to be SI as males. A total of 70.0% experienced FI, 4.7% were malnourished, and 34.7% were at high social risk. Women were most affected by FI, depression, SI and loneliness, Acute myocardial infraction, cerebrovascular accident, were contributors to risk of malnutrition. Lack of money and socialization contributed to staying in senior centers. Fi equated to social risk and diabetes as well.	N/A	N/A
Mehran et al. (42)	Fresh food Rx detected FI.	Reports on hospital patients’ intervention to address FI during COVID-19 pandemic. It is a non-experimental quantitative study. It screened for FI and connected those who qualified with an intervention. The intervention has not been implemented yet, so it was not measured	N/A	N/A
Hashemi et al. (43)	DASH reduced hypertension	Reports on DASH intervention. Both SI and FI were addressed through community senior centers and DASH-aligned congregate meal programs. DASH aligned meals along with monitoring blood pressure was found to be an effective intervention to control hypertension. Low income and FI pushed older adults to access organizations to assist with nutrition and food services. The mean of systolic blood pressure was found to decrease in the first month of the intervention.	Easy to implement into an existing meal program	Must have an existing meal program. Resistance to change.

SI, Social Isolation; FI, Food Insecurity; Rx, Medical Prescription; DASH, Dietary Approaches to Stop Hypertension; MNA, Mini Nutritional Assessment tool; NSENY, Nutrition Survey of the Elderly in New York State; FIES, Food Insecurity Experience Scale.

**Table 3:** Affinity matrix for thematic analysis.

Theme	References	Frequency of occurrence	Probability of occurrence
Social support	(38, 39), (40)*, (41)*, (43)	9	20.0%
Chronic disease status	(38)*, (39), (41)*, (42)	7	15.6%
Sex	(38)*, (40)*	5	11.1%
Socioeconomics	(38), (40)*, (41)	4	8.9%
Education	(38, 39, 43)	3	6.7%
Functional status	(39)*	3	6.7%
Food availability	(39, 41)	2	4.4%
Age	(38)*	2	4.4%
Marital status	(38)*	2	4.4%
Food insecurity paradox	(39, 41)	2	4.4%
Food assistance programs	(41, 42)	2	4.4%
Finance	(38)	1	2.2%
Medications	(38)	1	2.2%
Alcohol consumption	(40)	1	2.2%
Malnutrition risk	(41)	1	2.2%

*Multiple observations in the same article.

### 3.3 Risk of bias in studies and reporting of biases

The Johns Hopkins Nursing Evidence-Based Practice (JHNEBP) quality assessment tool (45) was used to evaluate bias and the overall quality of each study. The JHNEBP tool classifies strength of evidence in the following categories: Level I include randomized controlled trials; Level II include quasi-experimental studies without randomization; and Level III include observational and qualitative studies. Levels IV and V, include expert opinions, which were excluded from this review. To minimize the influence of bias on our findings, instances of bias were grouped and briefly analyzed, as bias can affect interpretation and generally limit external validity (46). The most common biases identified were sample bias (38, 39, 41–43), selection bias (38–43), and an affective health bias (42). Four of the six studies (38, 41–43) lacked intervention, and none included a control group, which weakens internal validity. Selection and affective health biases further threaten internal validity, while sample bias impacts external validity. Overall, the methodological limitations of these studies reduce the generalizability of their findings to broader populations.

The quality assessments showed that five out of six (83%) articles were level III non-experimental or qualitative studies, or meta-analyses. The quality of evidence showed that all six studies were level A (High), because the results were consistent, and the sample sizes were adequate.

### 3.4 Additional analysis and certainty of evidence

Table 3 summarizes the results of the observed thematic analysis: 11 themes and four individual observations were identified by the reviewers for a total of 45 occurrences in the literature.

In Table 3, the authors presented the descriptive statistics of the themes used in the reviewed studies. Of the 45 occurrences, nine (20%) identified social support as a factor (38–41, 43). Seven occurrences (15.6%) identified chronic disease status as a factor (38, 39, 41, 42). Five occurrences (11.1%) identified sex as a factor (40). Four occurrences (8.9%) identified socioeconomics as a factor (38, 40, 41). Three occurrences (6.7%) identified education as a factor (38, 39, 43). Three occurrences (6.7%) identified functional status as a factor. Physical limitations overall can lead to FI (39). Two occurrences (4.4%) identified food availability as a factor (38, 39). Two occurrences (4.4%) identified age as a factor (38). FI was identified to be highest in 60 + years individuals (38). Two occurrences (4.4%) identified the FI paradox as a factor (39, 41). Two occurrences (4.4%) identified food assistance programs as a factor (39, 42).

### 3.5 Interventions to address food insecurity and social isolation

Out of the six articles reviewed, only two interventions were identified (33%). The intervention of Fresh Food Rx linked patients facing FI to a food assistance program during the height of the COVID-19 pandemic through telehealth (42). The intervention of Dietary Approaches to Stop Hypertension (DASH) was used in community centers that served older adults (43). The DASH aligned meals found positive control of hypertension within one month (43).

## 4 Discussion

This systematic literature review examined six articles published over the past 10 years from five different countries, focusing on interventions addressing SI, FI, and malnutrition. The review reveals a significant gap in literature, as only six studies met the inclusion criteria. Notably, none of these studies employed a specific intervention model or utilized randomized controlled trials (RCTs), highlighting the limited evidence base in this area. The studies primarily relied on descriptive statistics and inferential analyses, including logistic regression and pre-test/post-test designs. The pre-post-test study consisted of a nutritional intervention to address blood pressure, FI, SI, and malnutrition using Dietary-Approach to-Stop-Hypertension (DASH) -based diet congregate meal program in community senior centers in New York City. The major objective of this intervention was to assess the effectiveness of DASH diet in lowering systolic blood pressure. The intervention was effective in lowering systolic blood pressure among DASH participants. Moreover, compared with individuals who did not experience FI, those who experienced FI reported higher systolic blood pressure (43).

Some validated instruments such as the Mini Nutritional Assessment survey was used to measure malnutrition levels, the Food Insecurity Experience Scale were used to assess FI, and Gijon’s social-familiar evaluation scale was used to measure social risk. These studies were conducted in different countries including Turkey (1 study) (38), Mexico (1 study) (39), Japan (1 study) (40), Portugal (1 study) (41), and the United States (2 studies) (42, 43). One of the articles from the U.S. was limited as it was a letter to the editor regarding an intervention to address FI among hospitalized patients during COVID-19 pandemic (42). The major findings from our review show that social factors and health status are the factors mostly associated with FI and malnutrition among older adults.

The social factors associated with FI consisted of being female, lack of money including pension and/or social security, lack of socialization, living alone loneliness, high level of social risk, low education level, and lack of access to community food programs (39, 41). The health-related factors associated with FI consisted of the presence of diabetes, depression, and physical impairment (41).

The social factors associated with malnutrition or the risk of malnutrition consisted of older age (≥ 70), being single, being a woman, being lonely and eating alone (not eating together with peers), poverty, and dealing with FI (38, 40, 41). The health status associated with malnutrition and/or risk of malnutrition consisted of poor health status and presence of chronic and acute diseases (depression, presence of orthopedic disability, acute myocardial infarction, and cerebrovascular accident) (38, 41).

None of the reviewed studies specifically targeted SI; however, by addressing FI and malnutrition, they implicitly engaged with aspects of SI. Although we anticipated identifying app-based food service interventions designed to address both FI and SI, none of the six studies employed mobile applications as a tool for meal delivery or social support. Most existing studies are cross-sectional and more appropriate for scoping reviews. Research on technology-based solutions for FI or SI is also limited, likely due to the complex challenges in public health, markets, and supply chains. An interdisciplinary approach combining public health, economics, and technology could help develop more effective, integrated interventions.

This systematic review has several limitations. First, although we queried four databases to reduce sampling bias, this approach yielded 138 duplicate articles, potentially reflecting overlapping coverage rather than expanding the diversity of sources. Second, the generalizability of the findings is limited. None of the studies were conducted across multiple countries, introducing selection bias and limiting global applicability. Furthermore, within-country generalizability is also constrained, as the study samples were not representative of the broader older adult populations in their respective nations. Third, five of the six studies exhibited sampling bias, with a predominance of female participants. Finally, methodological limitations, particularly the absence of randomized controlled trials (RCTs), mixed methods approach, and longitudinal data pose significant concerns regarding internal and external validity. These limitations restrict the extent to which the reviewed interventions can be confidently applied to the older adult populations.

Although our systematic review is based on six articles, it is a valuable review because it underscores the need for gold standards in research, randomized controlled trials (RCTs) using robust experiments to assess the effectiveness of interventions to address SI, FI, or malnutrition affecting the QoL of older adults. While RCTs focus on specific outcomes they may not capture the overall impact of an intervention, particularly where existing confounding variables like human behavior require holistic examination. Observational studies or non-randomized controlled trials in the form of cohort studies, case studies, or longitudinal research provide such insights into real-world applicability and scalability.

A notable gap in literature remains, as many technology-based interventions report only short-term impacts and are often tested on homogenous groups, limiting their relevance to diverse populations (47). Future research should employ longitudinal designs to evaluate the sustained effects of these interventions on FI and SI over time. Moreover, existing studies frequently neglect key behavioral and psychosocial indicators—such as social connectedness, mental health, and self-efficacy—that are closely tied to both challenges (48). Using validated scales for depression, loneliness, and perceived social support alongside food security measures would allow for a more comprehensive understanding of outcomes (49). Additionally, assessing technological barriers such as device availability, internet access, and user interface challenges is essential. Current evaluations often rely heavily on quantitative metrics like app usage or survey data, missing the nuanced, long-term behavioral shifts that digital interventions may produce (50). Comparative analysis of various approaches such as mobile apps, online forums, and telemedicine can further inform best practices and guide the integration of technology into public health strategies to more effectively and equitably address the intertwined issues of FI and SI.

Digital literacy also plays a vital role in addressing the overlapping challenges of SI and FI, in older adults, but significant barriers remain in the form of limited digital proficiency in older adults. Many lack the knowledge to navigate essential online platforms for food assistance programs like SNAP or WIC and cannot easily participate in telehealth services or virtual social activities (51). Cost is another major obstacle, as fixed incomes for older adults often make internet service, devices, and up-to-date technology unaffordable (52). Moreover, physical limitations, such as impaired vision or reduced mobility, and the absence of reliable broadband in rural or underserved areas further restrict digital access (53). To address these challenges, community-based initiatives have begun integrating food engagement with digital support. Programs such as virtual communal meals, online cooking classes, and recipe sharing platforms promote *commensality,* the shared experience of eating together, which enhances emotional and social well-being. Some holistic approaches that foster digital inclusion while meeting both nutritional and social needs include Commons Table, senior center tech workshops, library digital training, and device donation programs. However, large-scale technological solutions often fall short in addressing the specific needs of marginalized or underserved populations.

As the market for food production and distribution is often fragmented, with many players in the food supply chain with myriad interest; cost, accessibility, or infrastructure, particularly in low-resource settings, makes it difficult for developing advanced technology or food distribution innovations. Traditionally, public health research has focused on understanding the social, behavioral, and epidemiological aspects of FI and malnutrition, often relying on policy interventions or community-based solutions (54). Technologies that aim to address these issues may need to manage sensitive personal data, such as health and nutrition information. This raises concerns about privacy, consent, and ethical considerations, which could deter both research and technological development in this area.

## 5 Conclusion

FI, SI, and malnutrition are frequently associated with poorer outcomes in older adults and highlight the need for further research to clarify causal pathways and the extent of impact. Based on this systematic review, there has been a lack of interventions addressing these three issues simultaneously. Moreover, there is a need for conducting a scoping review that synthesizes information about the barriers and facilitators to improving food security and decreasing SI in older adults. A scoping review might suggest factors for consideration in developing potential interventions and by using a broader search criterion to capture all relevant information. Additionally, while technology could be leveraged to facilitate food delivery, to our knowledge, no app-based interventions have been published. We urge more technology-driven solutions to address these key challenges faced by older adults.

## Data Availability

The original contributions presented in the study are included in the article/supplementary material, further inquiries can be directed to the corresponding author.
